# Contemporary Techniques in Femoral Osteoplasty

**DOI:** 10.5435/JAAOSGlobal-D-24-00276

**Published:** 2025-08-28

**Authors:** J. Brett Goodloe, Vaibhav R. Tadepalli, F. Winston Gwathmey, Meghan W. Richardson, Charles A. Su

**Affiliations:** From the Department of Orthopedic Surgery, Sports Medicine Division, Virginia Commonwealth University, Richmond, VA (Dr. Goodloe and Dr. Richardson), and the Department of Orthopedic Surgery, Sports Medicine Division, University of Virginia, Charlottesville, VA (Dr. Tadepalli, Dr. Gwathmey, and Dr. Su).

## Abstract

Hip arthroscopy has a steep learning curve with femoral osteoplasty being one of the most challenging technical aspects of the procedure. The authors discuss a methodical system of preoperative templating and intraoperative fluoroscopic evaluation to ensure adequacy of resection and correction of impingement. There have been multiple methods developed to help aid surgeons in intraoperative decision making during cam correction. Using the available techniques may help limit complications and revision arthroscopic procedures, and understanding the nuances and limitations of each method will be paramount for the success of hip arthroscopists when addressing cam morphology. Future studies comparing long-term outcomes of these various resection techniques will be important in guiding our understanding of femoroacetabular impingement management.

The utilization of hip arthroscopy in the management of hip-related pathology continues to evolve and expand.^1^ Most commonly, hip arthroscopy is used for the treatment of femoroacetabular impingement (FAI) syndrome.^2^ Hip arthroscopy seems to be favorable for the appropriate FAI pathology when compared with open approaches (Campoamor Gonzalez, 2020 #69). From 2006 to 2010, there was a greater than 600% increase in the overall incidence of hip arthroscopy procedures among surgeons performing American Board of Orthopedic Surgeons Part II Board Examination (ABOS).^3^ Many fellowship programs incorporate hip arthroscopy training, and therefore, many young surgeons are performing hip arthroscopy early in their careers.^4^ With a youthful generation of surgeons attempting to master a technically demanding surgery, many researchers have investigated the steep learning curve of hip arthroscopy.^5-7^ Mehta et al^6^ described a long learning curve with 519 cases separating high-volume surgeons with the lowest complication profile in hip arthroscopy.

One of the most challenging aspects of arthroscopic management of FAI syndrome is the femoral osteoplasty.^8^ Translating two-dimensional radiographs into three-dimensional osseous corrections can be one of the most challenging tasks for a young hip arthroscopist.^5,6^ In addition to it being challenging, it is also very important. Most of the hip arthroscopists label the cam correction as the most crucial component of FAI management, leading to successful outcomes.^8^ In a systematic review of revision hip arthroscopic procedures, residual FAI attributed to femoral pathology was detected in more than 70% of patients.^9^ In addition, underresection or ignorance for the cam lesion has been associated with chondral damage and the potential development of an arthritic hip.^10,11^ Finally, a 15-year follow-up study showed notable improvements in patient-reported outcomes and THA-free survivorship in patients who underwent femoral osteoplasty as opposed to hip arthroscopy and labral repair alone (Nepple, 2022 #68).

As the number of primary hip arthroscopy procedures continues to rise, revision rates are similarly increasing; a 2021 database study of more than 15,000 patients found a 19% revision rate with 15.1% of patients undergoing a revision hip arthroscopy.^12^ Previous literature reports that incomplete or inadequate femoral cam correction has been one of the leading causes for revision hip arthroscopy.^13-15^ Cam lesions have been historically defined by using the alpha angle, which essentially measures asphericity of the femoral head commonly located along the anterolateral femoral neck.^16^ Using a burring device, a surgical resection of the cam lesion is performed. However, intraoperative assessment of optimal resection is challenging and may result in overresection or underresection.

Although historically underresection has been the bigger issue, there is now mounting literature discussing overresection as well.^17^ Overresection can lead to fractures of the femoral neck, disruption of the labral seal, hip instability, and new mechanical symptoms for patients.^18,19^ The consequences of overresection are potentially unsalvageable as well. Overresection can lead to loss of the labral-femoral head suction seal that helps maintain intra-articular pressures and the appropriate biomechanical environment for cartilage health.^19,20^ A 2018 study by Mansor et al^17^ found inferior outcomes in an overresection subgroup of patients when compared with underresected group within their population.

So how do young surgeons get it right? Undoubtedly, thorough intraoperative evaluation is necessary to confirm bony resection adequacy and rule out persistent bony impingement. Preoperative imaging can be compared with intraoperative fluoroscopy to evaluate completeness of the femoroplasty.^21^ Ross et al^22^ described six fluoroscopic views that can reliably detect cam deformity and provide a surgeons with a consistent evaluation of cam deformity intraoperatively. In recent years, there have been multiple methods described to guide and assist in creating a spherical femoral head and restoring the normal head-neck offset.^23-26^ This review aims to compile commonly used techniques for femoral osteoplasty and discuss the differences between them. The authors posit that the ideal method for guiding resection adequacy has not yet been discovered, but the knowledge of each available technique should help minimize risk and potential complications for the young practicing hip arthroscopist.

## Basics to Femoral Osteoplasty

Successful hip arthroscopy begins with appropriate patient positioning. The patient is placed in a supine position on a traction-type table with or without a post device. Obtaining fluoroscopic images before beginning the surgery ensures appropriate patient positioning and c-arm positioning. A full evaluation of each planned intraoperative image before beginning the case gives the surgeon confidence for being able to obtain each fluoroscopic image intraoperatively. General anesthesia and full muscle relaxation is often essential for central compartment evaluation and labral repair, but less pertinent for femoral osteoplasty as traction is often released during this part of the procedure.

Following the diagnostic arthroscopy and central compartment management, the peripheral compartment is entered, and the cam lesion is evaluated. Surgeons frequently use either an interportal capsulotomy or a T capsulotomy, which is dependent on many factors, including surgeon's preference, capsular mobility, size of cam lesion, and location of the cam lesion. Frequently capsular traction sutures are used to improve visualization as well. The capsulotomy chosen should enable the surgeon to adequately visualize and address the femoral sided impingement. Once visualization is established, a systematic approach to osteoplasty with the burr is used to perform bony resection. Most surgeons recommend starting medially and distally and then working proximally and laterally and alternating portals as needed for global femoral resection safely (Chow, 2014 #70). Next, we will present several arthroscopic surgical techniques for executing femoral osteoplasty.

## Alpha Angle Guided Resections

The alpha angle is the most commonly used radiographic measurement in evaluation of FAI and has frequently been used to assess the adequacy of femoral osteoplasty.^26-28^ The alpha angle serves as an useful parameter for quantifying cam lesions and are usually defined as greater than 55°.^29^ The alpha angle is measured on the Dunn lateral radiograph by placing a circle of best fit over the outline of the femoral head. A line is drawn from the first point on the femoral head-neck junction, which is outside the circle to the center of rotation of the femoral head. A second line is drawn from the center of the femoral head to the midline of the femoral neck, and the angle between these two lines is measured.^30^ During arthroscopy, there is a theoretical goal of decreasing the alpha angle to within normal limits. There is debate among the literature whether postoperative alpha angles correlate with patient-reported outcomes. A systematic review published in 2014 assessed the relationship between alpha angle correction and patient outcomes and concluded that alpha angle correction to less than 55° would result in improved outcomes.^31^ Alpha angle correction has also been found to be predictive of return to sport with greater correction correlating with higher return to sport and outcome measures in athletes.^32,33^ However, Briggs et al published conflicting results in 2017. Their group found no statistically significant differences between patient-reported outcome scores at five years in relation to alpha angle correction below 55°.^34^

**Figure 1:**
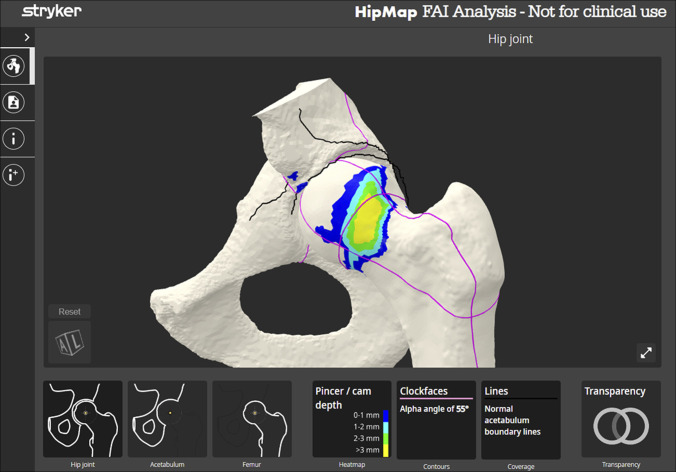
Image showing Stryker Hip Map Software using alpha angle in a three-dimensional model to demonstrate depth of resection required.

Recently, a complex navigation system using alpha angle has allowed surgeons to make intraoperative decisions to determine degree of alpha angle correction. The navigation system (Hip-Check; Stryker) allows for simultaneous measurement of the alpha angle on two-dimensional images ensuring adequate correction. Fluoroscopic images are obtained in a sequential manner similar to the six fluoroscopic images proposed by Ross et al.^22^ Following the acquisition of the images, alpha angles are calculated, and resection values are determined and monitored during the femoral osteoplasty. The navigation system can then measure postresection alpha angles to quantify the degree of correction on each fluoroscopic image (Figure 1).

There is limited comparative data available on the utility of computer navigation for femoral osteoplasty. Looney et al^35^ quantitatively compared computer navigation cam resection with traditional unguided resections in a retrospective review and showed comparable accuracy and validated accuracy and adequacy of femoral osteoplasty compared with traditional techniques. Additional research will need to be conducted to determine how these systems may affect patient-reported outcomes.

## Dynamic Hip Examination for Cam Morphology

**Figure 2:**
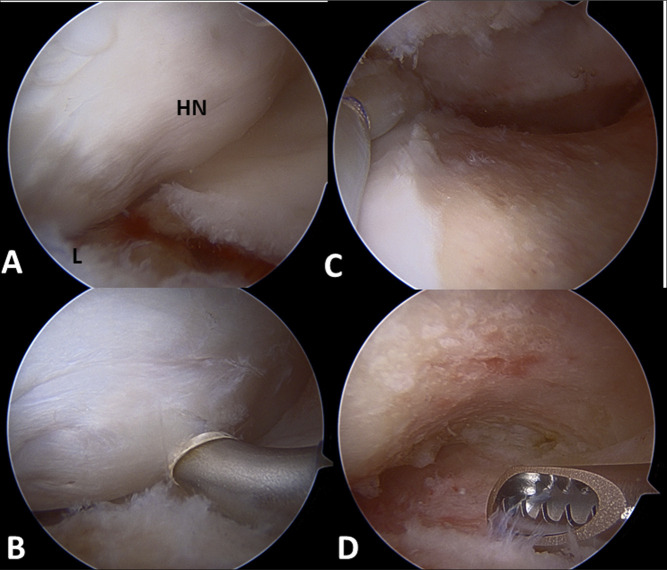
Arthroscopic view during dynamic hip examination. **A**, Dynamic hip examination in neutral abduction and flexion showing head-neck junction, as it approaches the repaired labrum (L). **B**, Dynamic hip examination in 45° of abduction with better visualization of the cam lesion shown by the RF. **C**, Anterior impingement test in neutral abduction and flexion showing no cam lesion on the head-neck junction following resection. **D**, Evaluation of the head-neck junction in 45° of abduction following resection. RF = Radiofrequency.

This technique, as described by Locks et al, is a purely arthroscopic technique performed with or without the use of fluoroscopic guidance (Figure 2).^23^ The dynamic examination is done to determine a sufficiency osteoplasty addressing impingement on an individualized basis. The authors of this technique propose that each patient has specific movements required for activity that are considered “motion at risk,” which should be assessed individually for each case. When no residual impingement is detected on dynamic examination, the femoral osteoplasty is considered adequate.

The examination begins after central compartment work is completed. Dynamic examination first focuses on the contact point with the labrum that elicits the greatest labral displacement. Removal of the foot from the traction boot or foot pad may allow for increasing hip motion and a more thorough assessment. The first maneuver done is taking the hip from full extension into flexion in neutral abduction. The authors have found that suction seal is often lost due to cam impingement around 70° of hip flexion. This suction seal can be re-established with appropriate cam resection. The hip is then assessed with varying degrees of abduction at both extension and flexion assessing superolateral impingement. For anterior FAI, the hip is then placed in flexion and maximal internal rotation.

For the femoral osteoplasty, the authors recommend at least a 15-mm distance from the proximal limit of the resection and the chondrolabral junction with the distal limit being the zona orbicularis. The medial and lateral limits are defined by the Weitbrecht ligament and the lateral retinacular vessels, respectively. Dynamic hip examinations are repeated until adequate resection is achieved, and there is no further evidence of levering on the cam lesion.^23^

## Perfectly Spherical Femoroplasty

**Figure 3:**
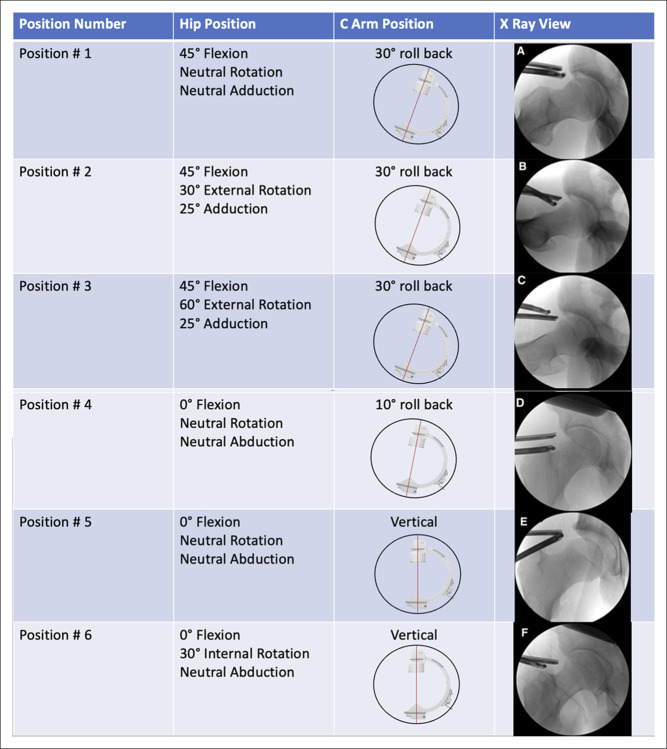
Image detailing the six positions described by Lall et al to evaluate the adequacy of femoroplasty during hip arthroscopy.

**Figure 4:**
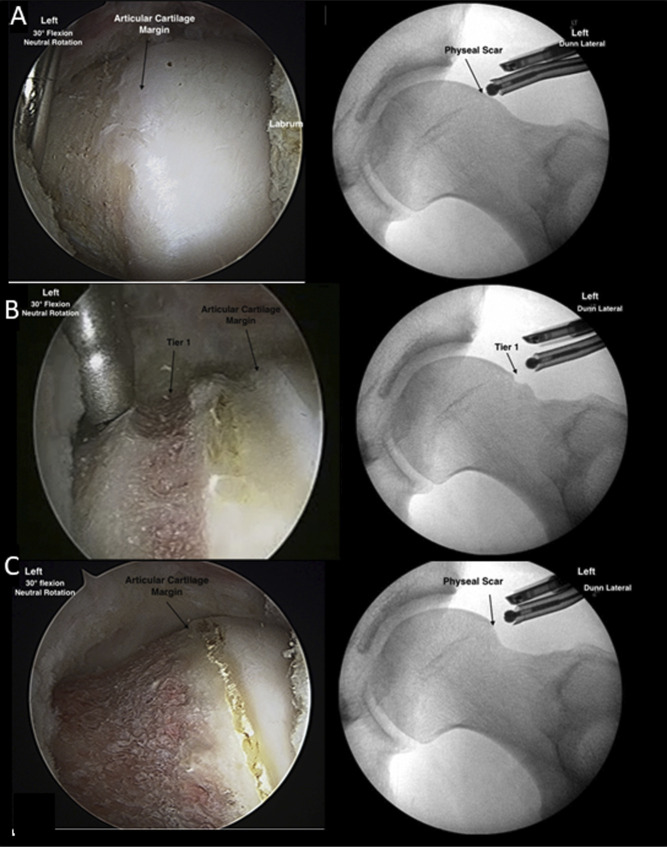
Image detailing arthroscopic and corresponding radiograph details of the two-tiered resection. **A**, Image demonstrating the preresection arthroscopic and fluoroscopic images in 30° of flexion and neutral rotation. **B**, The first tier of the resection demonstrating 1.5 burr lengths from the articular margin (**C**) Image demonstrating completion of the second tier of the resection and smoothing. Reproduced from Haase et al 2021 with permission from Journal of Arthroscopic Techniques.

As hip arthroscopists become more experienced, the aim of surgical correction of femoral impingement often shifts to creation of a perfectly spherical femoroplasty (Figures 3 and 4). Per Lall et al,^36^ achieving a perfectly spherical femoroplasty, uses six positions of the hip and c-arm to allow for accurate resection. The authors recommend starting in 45° of flexion and neutral rotation with the c-arm position of −30°. This positioning allows for fluoroscopic visualization of the anterolateral zone from 12:30 to 1:30 o clock. The surgeon then marks the proximal extent of the osteoplasty with electrocautery. This location is based on what is needed to optimize the alpha angle. Using an arthroscopic burr, more bone is resected distally than proximally to maintain convexity. This resection technique is repeated in each of the six positions.

For position 2, the surgical extremity is placed in 60° of flexion, 30° of external rotation, and 25° of adduction with the c-arm position maintained at −30° of cantilever. After resection is completed, the surgical extremity is placed in position 3. Transitioning to position 3 entails externally rotating the hip 30° extra to a position of 60° external rotation. These two positions allow for visualization of the anteromedial aspect of the femoral head-neck junction.

Positions 4, 5, and 6 allow for fluoroscopic guidance for the lateral and posterolateral zones of the femoral head-neck junction. The surgical extremity is placed in 0° of hip flexion with neutral rotation as well. For position 4, the c-arm is placed at 0° tilt, −10° cantilever. For position 5, the c-arm is repositioned to 0° cantilever, which allows for more posterior visualization of the femoral head/neck junction. Finally, position 6 is achieved by 30° of internal rotation of the surgical extremity, leaving the c-arm in the 0° tilt and 0° cantilever position. Following a measured resection through each position, a final fluoroscopic and dynamic examination is done to ensure a perfectly spherical head resection.^36^

## Two-Tiered Resection

**Figure 5:**
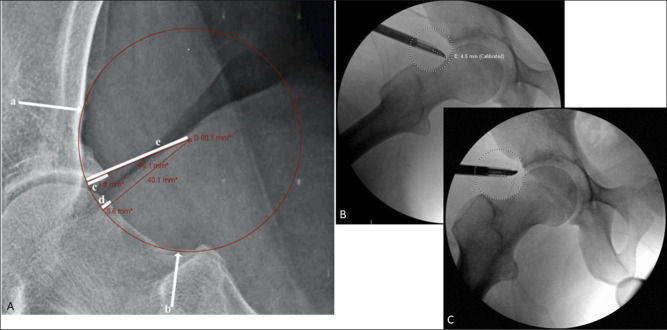
**A**, Image demonstrating the preoperative template for a patient on a Dunn lateral radiograph using the femoroacetabular impingement resection arc technique. The best fit circle is used to lay tangent to the anterior inferior iliac spine (indicated with the “a” arrow) and the superolateral femoral neck base (indicated with the “b” arrow). Resection depth is measured on the acetabular side (**C**) and the femoral side (**D**) and measured as shown in the figure. **B**, The intraoperative recreation of a preoperative plan on a separate patient showing area of intended resection within the arc of the circle. **C**, Post resection Dunn lateral radiograph showing the patient demonstrated in (**B**). Reproduced from Matache et al 2021 with permission from Journal of Arthroscopic Techniques.

As described by Haase et al, the two-tiered resection technique describes an additional method for achieving the perfectly spherical femoroplasty (Figure 5). This is an intraoperative tool to create a rounded head with symmetrical head-neck junction to the beta angle and achieve appropriate head-neck offset.^25^ The preoperative template is created using a Dunn lateral view to evaluate the correction beginning at the physeal scar to create a rounded head and remove the cam lesion distally. The authors propose beginning cam resection with the hip in 30° of flexion and neutral rotation. Fluoroscopic localization of the physeal scar with electrocautery is done before beginning resection to mark the proximal extent of the resection. The location of the proximal resection is found a few millimeters proximal to the insertion of the retinacular vessels as well. The authors describe the tier one resection as deepening resection proximally to the appropriate depth of head-neck offset, which allows for initial restoration of the head-neck offset. This technique also prevents overresection by setting the proximal depth first. Next, utilization of the traditional perpendicular view and the “up the neck” view allow for transitioning and contouring of the second tier to the initial set depth.^37^ Tier two does not affect the sphericity of the head but solely blends the head neck offset. The potential pitfalls of this technique include too proximal of a start point that may not allow for spherical femoral head creation and overzealous resection of the proximal start point, resulting in loss of suction seal. Whether these techniques to create a more spherical femoral osteoplasty affect patient-reported outcomes and functional scores following hip arthroscopy remain to be seen.

## The Femoroacetabular Impingement Resection Arc

As described by Matache et al, the FAI resection arc is a preoperative tool used on a 45° Dunn view radiograph to help determine a resection depth for a cam lesion. A best fit circle is used to incorporate the inferior aspect of the anterior inferior iliac spine and superolateral femoral neck base. This concept is comparable to Shenton line.^38^ This circle will create an impingement resection arc, which can be measured. Intraoperatively, once central compartment work is completed, the surgical hip is flexed to 45° and abducted to 20° to obtain an intraoperative Dunn. The cam resection is performed based on preoperative templating in a stepwise fashion from medial to lateral with progressive extension of the hip. Comparative fluoroscopic images are used to compare the FAIR arc template. This technique not only measures the resection of the cam lesion but also addresses acetabular morphology. Kaplan et al used the FAIR arc as a tool for measuring the maximal radial distance (MRD) of the cam lesion in the preoperative and postoperative settings following surgical management of FAI. The authors concluded that at 2-year follow-up, a reduction of the MRD below 3.15 mm resulted in improved outcomes when compared with patients with a residual cam MRD greater than 3.15 mm.^39^ Therefore, this technique may also provide quantification for measured resection, which could potentially be useful for surgeons seeking resection adequacy.

## Summary

Hip arthroscopy has a steep learning curve with femoral osteoplasty being one of the most challenging technical aspects of the procedure. The authors recommend that each surgeon create a methodical system of preoperative templating and intraoperative fluoroscopic evaluation to ensure adequacy of resection and correction of impingement. There have been multiple methods developed to help aid surgeons in intraoperative decision making during cam correction. Using the available techniques may help limit complications and revision arthroscopic procedures, and understanding the nuances and limitations of each method will be paramount for the success of hip arthroscopists when addressing cam morphology. Future studies comparing long-term outcomes of these various resection techniques will be important in guiding our understanding of FAI management.
